# The Effect of Various Mixing Techniques on the Push-Out Bond Strength of Calcium Enriched Mixture

**Published:** 2016-06

**Authors:** Nooshin Sadat Shojaee, Alireza Adl, Fereshte Sobhnamayan, Fatima Vasei

**Affiliations:** 1 Dept. of Endodontics, School of Dentistry, Shiraz University of Medical Sciences, Shiraz, Iran.; 2 Dept. of Endodontics and Biomaterials Research Center, School of Dentistry, Shiraz University of Medical Sciences, Shiraz, Iran.; 3 Undergraduate Student, School of Dentistry, Shiraz University of Medical Sciences, Shiraz, Iran.

**Keywords:** Calcium Enriched Mixture, Push-out Bond Strength, Mixing Method, Ultrasonic

## Abstract

**Statement of the Problem:**

Correct proportioning and mixing are essential to ensure cements attain their optimum physical properties.

**Purpose:**

The aim of this experimental study was to evaluate the influence of various mixing techniques including manual, mechanical mixing, and ultrasonic vibration on push-out bond strength of calcium enriched mixture (CEM).

**Materials and Method:**

Ninety 2-mm-thick dentin disks were prepared from single-rooted human teeth and filled with CEM mixed with manual, trituration, or ultrasonic methods. Push-out bond strength values of the specimens were measured by a universal testing machine after 3 and 21 days. The samples were then examined under a stereomicroscope at 40× magnification to determine the nature of bond failure. Data were analyzed by Kruskal-Wallis and Mann-Whitney test. (*p*< 0.05)

**Results:**

The highest (7.59 MPa) and lowest (4.01 MPa) bond strength values were recorded in conventional method (after 21 days) and trituration method (after 3 days), respectively. There was no statistically significant difference between the three techniques in 3 and 21 days.

**Conclusion:**

According to the results, various mixing techniques had no effect on the push-out bond strength of CEM cement.

## Introduction

Many types of dental cements are available as powder and liquid that should be mixed before application. Correct proportioning and mixing are essential to ensure that the cements attain their optimum physical properties.[1] Encapsulating along with trituration in comparison to manual mixing has the potential to reduce air spaces between adjacent particles. It results in a more thorough wetting of the powder particles and improves the unification of the resultant paste.[2]

Whilst trituration uses conventional mechanical energy, there might be a potential for ultrasonic energy to be more effective. Ultrasonic vibration has a dispersing effect on the particles of materials, which frequently cluster together. Ultrasonic treatment has been reported to be effective in increasing the compressive strength,[3-4] tensile bond strength,[5-6] and hardness[7] of glass ionomer cements.

Little information is available on the effect of various mixing techniques on the physical properties of mineral trioxide aggregate-like (MTA) materials. Nekoofar *et al.* compared ultrasonication, trituration, and manual mixing and concluded that the application of ultrasonic energy to MTA produced a significantly higher surface microhardness value.[8] On the other hand, Shahi *et al.* reported that different mixing methods had no significant effect on the push-out bond strength of white MTA.[9] Basturk *et al.*, however showed that mechanical mixing of encapsulated MTA resulted in higher compressive strength values than those mixed manually.[10]

Calcium enriched mixture (CEM) cement was introduced in 2008 with similar clinical application to MTA but different chemical compositions.[11-12] CEM is tooth-colored water-based cement which consists of calcium oxide, calcium phosphate, calcium carbonate, calcium silicate, calcium sulphate, calcium hydroxide and calcium chloride.[13] This cement exhibited favourable results in regard to biocompatibility, antibacterial effect, and sealing properties.[13-18]

There is no information about the effect of mixing techniques on the push-out bond strength of CEM cement. Therefore, the purpose of this study was to evaluate the effect of various mixing techniques including ultrasonic vibration, trituration, and manual method on push-out bond strength of CEM cement. 

## Materials and Method

Sixty freshly extracted human teeth including single rooted mandibular premolars or maxillary incisors that were either intact or contained only small carious lesion were used in this study. Teeth with cracks or internal resorption were excluded from the study. After removing the crowns by using a diamond disk, the middle thirds of the teeth were sectioned perpendicular to the root long axis to obtain 90 dentin disks with the thickness of 2±0.2 mm. A diamond saw microtome (Mecatom T180; Presi SA, Angonnes, France) was used to obtain root dentin slices. The internal disk canals space was enlarged with Gates Glidden burs (Dentsply Maillefer; Ballaigues, Switzerland) sizes 2 to 5 to achieve a standard diameter of 1.3 mm.[9] The root sections were immersed in 17% EDTA (ethylenediaminetetraacetic acid) (Asia Chemi Teb; Tehran, Iran), and then in 2.5% sodium hypochlorite (Pakshooma; Tehran, Iran) each for three minutes to remove the smear layer. They were, then, washed with distilled water and dried.[9] The root sections were randomly divided into 6 groups (n=15), and the lumens were filled with CEM cement (BioniqueDent; Tehran, Iran) as following.

In groups 1 and 4 the CEM cement was mixed with conventional method; in groups 2 and 5, the CEM cement was mixed with trituration in an amalgamator (Farazmehr; Esfahan, Iran) at the speed of 4500 revolutions/min for 30 second (customized encapsulated CEM); in groups 3 and 6, the CEM cement was mixed with an ultrasonic tip (Ultradent Products; Inc., Logan, UT, USA). The CEM cements in all instances were mixed according to the manufacturer’s recommendations. The samples were wrapped in wet pieces of gauze and kept in an incubator (Mart Microbiology B. V.; Netherlands) at 37 ˚C and 95% humidity for 3 days (groups 1, 2, and 3) or 21 days (groups 4, 5, and 6). 

**Push-out test**

**Figure 1:**
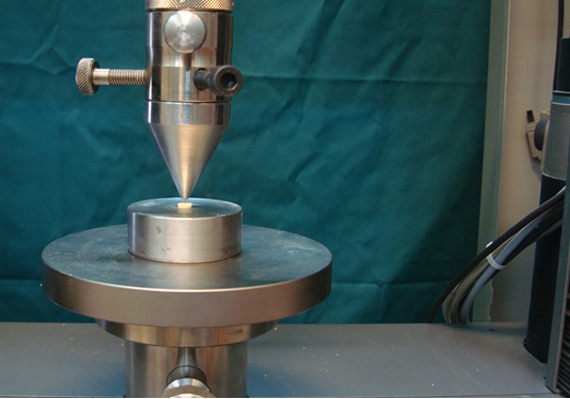
Universal testing machine

The push-out test was performed on the samples by using a universal testing machine (Zwick/Roell, Z050; Zwick/Roell, Ulm, Germany) (Figure 1).

The cured specimens were placed on a metal slab with a central hole and loaded with a 0.7-mm diameter cylindrical stainless steel plunger at a speed of 1mm/ min. The maximum load applied to the CEM cement at the time of dislodgement was registered in newton.

To express the bond strength in megapascals (MPa), the recorded values were divided by the adhesion surface area of CEM in mm^2^ calculated according to the following formula:

2πr × h, where π is the constant 3.14, r is the root canal radius, and h is the thickness of the root slice in millimetres.

The modes of bond failure were evaluated under the light microscope (Dino-light; Hsinchu, Taiwan) at 40× magnification. Each sample was categorized into one of the three failure modes as adhesive failure at the CEM and dentin interface, cohesive failure within the CEM, and mixed failure mode. The data were analyzed by using Kruskal-Wallis and Mann-Whitney test as post-hoc test. (*p*<0.05)

## Results

The means and standard deviations (SD) of the push-out bond strength of the groups are shown in Table 1.

**Table 1:** The Means and standard deviations of push-out bond strength of experimental groups

**Group**	**Mean)MPa)(SD)**
Group1 (Conv, 3days)	4.860±1.413
Group 2 (Trit ,3 days)	4.016±1.322
Group 3 (Ultra ,3days)	4.848±2.122
Group 4 (Conv, 21days)	7.598±5.062
Group 5 (Trit ,21 days)	4.548±4.485
Group 6 (Ultra, 21days)	5.104±3.872

Abbreviations: Conv: Conventional method, Ultra: Ultrasonic method, Trit: Trituration method

**Figure 2:**
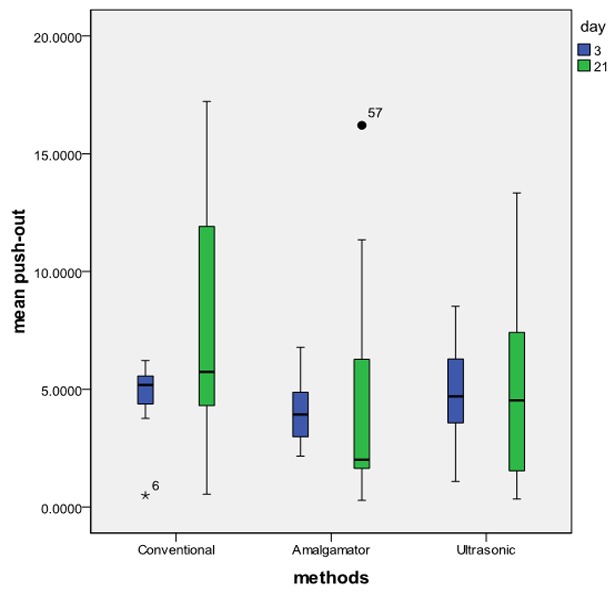
The mean of push-out bond strength values of CEM by 3 mixing methods

Although the groups that CEM was mixed with trituration (group 2 and 5) showed the lowest value of push-out bond strength, no statistically significant difference was found between the three techniques in 3 and 21 days (Figure 2).

**Figure 3:**
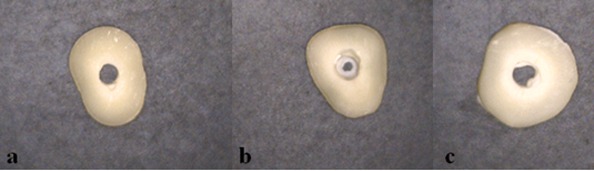
Modes of failure a: adhesive failure; note the clean canal wall. b: cohesive failure within CEM. c: mixed failure; note the MTA residual inside the canal.

Inspection of the samples revealed the bond strength to be predominantly cohesive for conventional technique but mixed for trituration and ultrasonic techniques. (Table 2 and Figure 3) (*p*<0.05)

**Table 2:** Percent of each mode of failure among the experimental groups

**Experimental Group**	**Cohesive** **(%)**	**Adhesive** **(%)**	**Mixed** **(%)**
Group1 (Conv, 3 days)	73.3	0	26.7
Group 2 (Amal, 3 days)	20	20	60
Group 3 (ultra, 3 days)	46.7	0	53.3
Group 4 (Conv, 21days)	40	13.3	46.7
Group 5 (Amal, 21 days)	0	20	80
Group 6 (Ultra, 21days)	20	33.3	46.7

## Discussion

In this study, push-out test was used to assess the bond strength between CEM cement and dentinal walls. Various methods have been described to evaluate the bonding quality of dental materials to dentin such as shear,[19-20] compressive,[4] tensile,[4, 6] flexural[22] and push-out bond strength.[9-10,22-23] Among them, push–out test has been shown to be efficient and reliable.[23]

A scanning electron microscopy (SEM) and energy dispersive X-ray analysis (EDXA) study demonstrated that in the presence of normal saline as a storage solution, hydroxyapatite crystals are formed and precipitated over the surface of CEM cement. The composition and structure of precipitated crystals were comparable with that of standard hydroxyapatite.[24] Therefore, in the present study, the root slices were wrapped in pieces of gauze soaked in normal saline.[25]

To achieve optimal properties, the particles of hydraulic cements should be thoroughly mixed with water. The mixing technique of cements is fundamental for producing effective contact between the powder particles and liquid and a final set material with optimal physical, chemical, and biological properties.[2] It has been reported that mixing methods have a significant effect on porosity and compressive strength of glass ionomer cements.[1, 26] Regarding MTA, it has been demonstrated that mechanical mixing enhanced the compressive strength[9] and ultrasonic vibration produces a significantly higher surface microhardness.[8] In addition to mixing techniques, root end preparation techniques have been shown to influence the bond of endodontic materials to dentinal walls. Shokouhinejad *et al.* evaluated the push-out bond strength of two root-end filling materials in root-end cavities prepared by Er,Cr:YSGG laser or ultrasonic technique and concluded that the bond strength of MTA and CEM to root-end cavities were comparable and higher in ultrasonically prepared cavities.[27]

In the present study, the influence of various mixing techniques on push–out strength of CEM cement was evaluated for the first time. The results showed that trituration method compared with manual and ultrasonic techniques results in lower push-out strength; however, no statistically significant difference was found between the three mixing techniques. 

Another study on the effect of different mixing techniques on the compressive strength showed that mechanical mixing with amalgamator increased the compressive strength of CEM cement.[28] The conflicting results of these two studies can be attributed to the fact that push-out test and compressive strength have different natures.

Interestingly, the studies on MTA showed similar results. While Shahi *et al.*[9] showed that different mixing methods had no significant effect on the push-out bond strength of MTA; Basturk *et al.*[9] reported that mechanical mixing of encapsulated MTA resulted in higher compressive strength value than those mixed manually.

Therefore, one may assume that different mixing methods have impact on the compressive strength, not on the push-out strength of hydraulic cements like MTA and CEM.

The results of the current study showed that there was no significant difference between the push out bond strength of similar groups in 3 and 21 days. This finding is in contrast with that of Rahimi *et al.*[29] who reported an increase in the bond strength of CEM cement from 24 hours to 7 days. The reason for the observed disagreement may be related to the different experimental set-up of the two studies. Gancedo-Caravia and Garcia-Barbero[30] showed that curing conditions do play an important role in the retention characteristics of MTA. Therefore, different studies with different curing condition should not be expected to have similar results.

In this study inspection of the samples revealed the bond strength to be predominantly cohesive for manual technique, but mixed for trituration and ultrasonic techniques. Therefore, it can be concluded that mixing method can affect the pattern of bond failure. Two separate studies with manual mixing technique reported cohesive bond failure as the predominant bond failure for CEM, which is in agreement with the results of the current study.[31-32]

It should mention that the mechanical tests are unable to reflect the clinical situation; hence, future studies are required to determine the effect of these techniques on the bond strength of material in clinical applications. 

## Conclusion

Within the limitation of this *in vitro* study, it can be concluded that different mixing techniques evaluated in this study have no effect on the push-out bond strength of the CEM cement
